# Association Between Preschoolers’ Specific Fine (But Not Gross) Motor Skills and Later Academic Competencies: Educational Implications

**DOI:** 10.3389/fpsyg.2020.01044

**Published:** 2020-06-05

**Authors:** Elena Escolano-Pérez, Maria Luisa Herrero-Nivela, José Luis Losada

**Affiliations:** ^1^Department of Psychology and Sociology, University of Zaragoza, Zaragoza, Spain; ^2^Department of Social Psychology and Quantitative Psychology, University of Barcelona, Barcelona, Spain

**Keywords:** motor skills, academic competencies, systematic observation, early childhood assessment, child development, learning, preschoolers, educational practice

## Abstract

Motor development is an inseparable component of cognitive development. So, to develop the mind, it is necessary to work the body. Therefore, Early Childhood Education curricula and the scientific literature emphasize the need to promote the development of motor skills during the 1st years of life. These skills are necessary for learning and subsequent academic performance. However, studies frequently offer only a partial view of these relationships. Few works have analyzed the specific relationships between different components of preschool gross and fine motor skills and subsequent performance on different academic competencies. Further, they present discrepant results. The aim of this study was to determinate which specific components of gross and fine motor skills assessed in Spanish students during the final year of Early Childhood Education (5 to 6-year-olds) were associated with different academic competencies assessed in the following academic year, when the students were in their 1st year of Primary Education. The final sample consisted of 38 Spanish students, aged 5. A mixed methods approach was used. It consisted of systematic observation to assess specific components of gross and fine motor skills when children were in the Early Childhood Education period, and selective methodology to evaluate their academic competencies (specifically in literacy and mathematics and overall), 1 year later, once in Primary Education. Multiple linear regression models were constructed using the computing language R to examine the association between motor skills and academic competencies. The results indicated that only the components of fine motor skills showed associations with academic competencies. The pattern of association varied when literacy and mathematics competencies were specifically and individually assessed and when overall academic competency was considered. The two assessed fine motor skills (Coordination and Integration) were associated with literacy competency (β = 0.344, *p* = 0.025; β = 0.349, *p* = 0.024, respectively) and overall academic competency (β = 0.267, *p* = 0.065; β = 0.493, *p* = 0.001, respectively). However, only Integration was associated with mathematics competency (β = 0.476, *p* = 0.002). The “Discussion” section focuses on the educational implications of these results and future research. It highlights the importance of early assessment of fine motor skills to identify students likely to present inadequate subsequent academic performance and the need to apply instruction and interventions tailored to the specific needs of each child.

## Introduction

Diverse development theories and numerous authors have highlighted the relationship existing between motor and cognitive development (Frick and Möhring, 2016). Decades ago, Wallon (1977) declared that children develop through movement. This development takes place “from the act to the thought” (Wallon, 1942), from the concrete to the abstract, from the action to the representation, from the physical to the cognitive. Piaget (1936, 1970, 1973) also suggested that bodily action prepares logical operations, since logic is based on the coordination of actions, prior to being formulated on the language plane. Thus, he established sensory-motor or practical intelligence as the base of verbal or reflexive intelligence. Pelicier et al. (1996) stated that motor and psychological functions are the two fundamental elements of human behavior. Initially, they develop together, later being specialized and differentiated, although they continue to be subject to reciprocal interactions (Adolph and Franchak, 2017; Kim et al., 2018).

The relationships between motor and cognitive aspects have been corroborated by empirical data based on different types of studies: (a) neuro-functional and neuro-anatomical studies: data resulting from functional magnetic resonance techniques reveal that motor and cognitive development follow a common extended development pattern, sharing anatomical areas that were previously considered to be specific to only one of the development types (Schmahmann, 2019). So currently, it is known that there is a clear connection between the brain areas involved in motor skills (mainly, the cerebellum) and those involved in cognitive skills (mainly, the pre-frontal cortex); and the development of both takes place simultaneously and is especially rapid over the 1st years of life, with a developmental peak occurring between 5 and 10 years of age (Ahnert et al., 2009; Haartsen et al., 2016; Leisman et al., 2016). Thus, both brain structures are active when carrying out certain motor or cognitive tasks. Other structures, such as basal ganglia, and certain neurotransmitters, such as dopamine, also appear to be involved in certain complex components of motor and cognitive performance (Diamond, 2000, 2007; Leisman et al., 2016; Jung et al., 2017). (b) Studies carried out on patients suffering from cerebral lesions and developmental disorders: individuals having cerebral lesions in the primary motor area or in the primary cognitive area often reveal deficits in both types of skills (Diamond, 2000, 2007; Rooijen et al., 2012). Likewise, many disorders exist in which motor problems are accompanied by learning difficulties or cognitive deficits, as is the case with the Developmental Coordination Disorder (DCD), Attention-Deficit and Hyperactivity Disorder (ADHD), or Autism Spectrum Disorder (ASD) (Blank, 2018; Lange, 2018; Scandurra et al., 2019). (c) Longitudinal studies carried out on normal populations: many longitudinal studies have found that the relationship between motor skills and cognitive development continues over the short, medium, and long term. So, motor skills acquired at a very early age may relate to cognitive abilities during childhood (Son and Meisels, 2006; Michel et al., 2016), adolescence (Cantell et al., 2003), and even adulthood (Kuh et al., 2006; Murray et al., 2006). This suggests that performing early motor development assessments may help to identify children having a probability of demonstrating poor academic performance and even adults who may have difficulties in entering the work force (Son and Meisels, 2006; Cameron et al., 2012, 2016; Roebers et al., 2014; Pitchford et al., 2016; Schmidt et al., 2017; Goodway et al., 2019).

In the field of Early Childhood Education (ECE), numerous studies have defended the idea that motor skills are associated with academic competencies and achievement (Grissmer et al., 2010; Cameron et al., 2012, 2016; Pitchford et al., 2016). However, upon analyzing their results, it may be found that the association between motor and academic achievement has yet to be well established in childhood. One of the issues that may explain this situation is based on the fact that motor skills and academic achievement are broad concepts. Most studies only focus on some of their components, offering a partial view of motor development and academic performance as well as their associations.

In motor development, two main types of skills have been traditionally considered: (1) Gross motor skills and (2) Fine motor skills (Grissmer et al., 2010; Bjorklund and Hernández, 2012; Gentier et al., 2013; Raisbeck and Diekfuss, 2015; van der Fels et al., 2015; Oberer et al., 2017; Haywood and Getchell, 2019). (1) Gross motor skills refer to actions of large muscle and postural groups; movements of the entire body or large body segments. They include specific skills: (1a) Locomotor skills: involving the coordination of the entire body, allowing for the movement of the body from one point in space to another, using body movement to achieve this. Locomotor skills include running, galloping, hopping, leaping, jumping, and sliding. (1b) Balance: this refers to the ability to hold a controlled position or posture during a specific task or activity. There are two types of balance: (i) Dynamic Balance refers to the ability to maintain a position during activities that require movement, such as walking. It is obtained when stability of the body is held during movement performance. (ii) Static Balance refers to the ability to maintain position during stationary tasks, such as standing or sitting. A task that is commonly used to assess this balance type is the ability to remain standing on only one leg. (1c) Object Control skills: movements in which the main action focuses on the handling of objects. It includes all tasks that involve the handling of objects (such as, for example, throwing, catching, hitting, absorbing, etc.), be it with the hands, feet or other objects. These skills may be separated into two sub-types: (i) Propulsive skills are those that involve sending an object away from the body (overhand throw and underhand roll, hitting a ball with a tennis racket, kick, etc.); (ii) Receptive skills involve receiving an object. They involve an absorption movement, that is, they serve to slow down a movement in order to handle it (stationary bounce, match) (Ulrich, 2000; Grissmer et al., 2010; Lopes et al., 2013; D’Hondt et al., 2014; Magistro et al., 2015; Rudd et al., 2015; Chang and Gu, 2018; Haywood and Getchell, 2019). (2) Fine motor skills involve the action of small muscle groups; precise movements of the hands, face and feet, such as, for example, the ability to use hands. Within this type of skills, two specific skill types may be differentiated: (2a) Fine Motor Coordination or Visual Motor Coordination: it refers to small muscle movements, but not to the integration of these muscle movements with other input, such as visual-spatial information, from the environment. It includes certain abilities such as finger dexterity, motor sequencing, and fine motor speed and accuracy. Some of the tasks that are commonly used to assess it are: tracing, finger tapping, imitative hand movements, building with blocks, threading beads, replacing pegs, moving coins from one place to another or inserting them in a slot (Davis and Matthews, 2010); (2b) Fine Motor Integration: it involves the organization of small muscle movements in the hand and fingers through the processing of visual stimuli. Visual information from the environment must be processed and integrated with fine motor movements (Sortor and Kulp, 2003). It relies more on synchronized hand-eye movements than Fine Motor Coordination. For its assessment, writing and copying tasks are carried out on shapes, letters or other stimuli (Grissmer et al., 2010; Cameron et al., 2012; Roebers et al., 2014; Jansen et al., 2015; van der Fels et al., 2015; Oberer et al., 2017; Chang and Gu, 2018). The development of gross and fine motor skills does not take place independently. For example, biped walking leaves the hands free, permitting new possibilities of action and representation (Oberer et al., 2017).

As described above, various components or specific skills are involved in each of the two main motor skills (gross and fine). However, the majority of studies analyze only one type of these main motor skills (gross or fine); and very few have considered their distinct specific components. Therefore, these may be considered partial studies. Other works have assessed the distinct specific gross and fine motor skills, but they are quite scarce (Oberer et al., 2017). And paradoxically, some of these later consider the specific gross and fine motor skills in a global sense, offering a sole score for each main type of motor skill (gross or fine) or even one single overall indicator for all of them. This implies a confused perspective and a lack of depth to the topic. In accordance with many other authors (Oberer et al., 2017; Schmidt et al., 2017), we highlight the need to operationalize the childhood motor skills through distinct specific gross and fine motor skills that suitably represent their multidimensional nature.

The same occurs with academic performance. As previously mentioned, this is also a very broad concept. However, some studies offer only one overall score for academic performance. This is a limitation, since, according to the scholastic curriculum, the academic competencies that are studied in schools and that should therefore be assessed, belong to diverse domains (Organization for Economic Co-Operation and Development, 2005; Education, Culture and Sports Ministry of the Spanish Government, 2015). Traditionally, studies considering various curricular aspects distinguish between literacy and mathematics achievement (Fernandes et al., 2016; Abdelkarim et al., 2017; Ribner et al., 2017). Literacy and mathematics are considered to be the core academic domains since well-developed competencies in these areas are critical for performance in other scholastic fields such as geography and history, and for success in the child’s subsequent studies. Children need these academic competencies in order to reach their full potential, thereby paving the road to a successful professional life (Organization for Economic Co-Operation and Development [OECD], 2016).

Since, generally speaking, studies have failed to collectively consider the distinct specific motor skills and the distinct academic competencies, there is a lack of conclusive data to affirm the extent to which each specific motor skill can be associated with academic performance in the distinct areas (Magistro et al., 2015; Veldman et al., 2019). Furthermore, of the few studies that have analyzed the different specific motor skills and academic competencies, it is difficult to reach conclusions as to the associations between them, since each study analyzes different motor skills and academic competencies, or operationalizes them in different ways; and they have been assessed in populations with distinct characteristics. All of these aspects contribute to the disparity of results in this area. Therefore, this is a complex area of study, filled with partial results and contradictory situations, making it difficult to reach consensual conclusions.

Ultimately, it is necessary to thoroughly and profoundly consider the potential existence of specific associations between the distinct components of motor skills and academic competencies in order to contribute to the children’s success. To do so, as mentioned previously and in accordance with other authors (York et al., 2015), it is necessary to collectively examine the specific gross and fine motor skills, as well as literacy and mathematics competencies (in addition to overall ones) in one study. However, this type of study is scarce and the results of the few that have been conducted are quite disparate.

This study has been carried out in an attempt to eliminate this gap. Its objective was to determinate which specific gross and fine motor skills, assessed in Spanish students in the last year of ECE (5–6 years), were associated with later academic competencies (specifically in both literacy and mathematics and overall competencies) assessed during the following academic year, when the students were in the first quarter of Primary Education.

Determining these associations in preschool children may help with the design and implementation of effective teaching and interventions to improve the specific motor skills that are most relevant for the subsequent academic competencies. This would promote the future academic success of students from a young age, helping to strengthen the country by ensuring the educational success of all of its inhabitants (Organization for Economic Co-Operation and Development [OECD], 2016).

## Materials and Methods

### Methodology and Design

We applied a mixed methods approach (Anguera et al., 2017, 2018b; Escolano-Pérez et al., 2019a, b) consisting of systematic observation to observe preschoolers’ motor skills and selective methodology to assess their academic competencies the following year.

We employed systematic observation to observe preschooler motor skills for several reasons: (1) The study was carried out in the school context of the participants, specifically, in their regular motor development sessions within their scholastic program. These motor development sessions make up a regular and necessary part of ECE (the educational stage at which the participants were), since some of the purposes of the same include the discovery of body and movement possibilities, the development of more voluntary motor activity in the children and the acquisition of progressive body control (Education and Science Ministry of Spanish Government, 2008). (2) Spanish education regulations (in addition to those of distinct international institutions and the scientific literature) indicate that during this scholastic phase, the assessment of student learning and development should be carried out mainly via direct and systematic observation (Education and Science Ministry of Spanish Government, 2008; Early Head Start National Resource Center, 2013; Otsuka and Jay, 2017). (3) This is coherent with the methodological requirements of systematic observation: having perceivable and regular behaviors in a natural setting (Shaughnessy et al., 2012; Portell et al., 2015a, b; Anguera et al., 2018a, b).

According to the observational designs described by Anguera (2001) and Anguera et al. (2011, 2018a), the study was carried out using a Nomothetic/Punctual/Multidimensional (N/P/M) design. Nomothetic refers to the observation of several different children, each of whom was observed individually. Punctual refers to the recording of the motor execution of each child in each activity of interest, in a single observation session. Multidimensional refers to the fact that more than one dimension of the participant’s response is taken into account; more precisely, different aspects of the child’s motor execution were observed with reference to specific gross and fine motor skills, in accordance with the theoretical proposal of several authors (Ulrich, 2000; Grissmer et al., 2010; Bjorklund and Hernández, 2012; Gentier et al., 2013; Lopes et al., 2013; D’Hondt et al., 2014; Jansen et al., 2015; Magistro et al., 2015; Raisbeck and Diekfuss, 2015; Rudd et al., 2015; van der Fels et al., 2015; Oberer et al., 2017; Chang and Gu, 2018; Haywood and Getchell, 2019). These dimensions of the participant’s response led to the *ad hoc* observation instrument that was designed.

Observation was non-participative and active, based on scientific criteria and characterized by total perceptibility. Direct observation of the film recorded was carried out (Bakeman and Quera, 2011; Shaughnessy et al., 2012; Anguera et al., 2018a, b).

For the assessment of the academic competencies 1 year later (during the first school year of Primary Education), a selective methodology was used. Specifically, the standardized PAIB-1 (*Test of basic instrumental aspects: Reading, writing and numeric concepts*; Galve-Manzano et al., 2009) instrument was administered. The assessment of academic competencies through a standardized instrument such as the PAIB-1 guaranteed a more objective, reliable, and valid assessment, as compared to the use of scores given by the teachers. These have been found to be less reliable, also leading to other problems such as a lack of comparability with other teachers or schools (Marzano, 2000; Organization for Economic Co-Operation and Development [OECD], 2012; Castejón et al., 2016).

### Participants

The study participants were selected intentionally. They were enrolled in a school that declared its interest in participating in a study that was directed by the first author of this manuscript. Thus, the studied sample was part of a larger research project.

The school was located in the center of a Spanish city. The students attending this school came from middle to upper socio-economic level families.

Inclusion criteria for the sample were: (1) being a student in the 3rd year of ECE (in Spain, this course year corresponds to an age of 5–6 years and is the last year in this non-mandatory school phase); (2) attendance at the targeted school since the 1st year of ECE (that is, since 3 years of age); (3) anticipating the continued study in this same school the following year (that is, intent to enroll in the first course year of Primary Education in the same school); (4) absence of the following disorders or risk factors: (a) birth weight < 2,000 g and/or gestational age <36 weeks or significant pre, peri-, or postnatal events; (b) medical/neurological conditions affecting growth, development, or cognition (e.g., seizure) and sensory deficits (e.g., vision or hearing loss); (c) neurodevelopmental disorders (e.g., ASD, ADHD, and language disorder); (d) genetic conditions or syndromes; and (e) a first-degree relative with schizophrenia, bipolar disorder, or related disorders; and (5) an adequate IQ for their chronological age.

The assessed information to ensure compliance with criteria 1 (being a student in the 3rd year of ECE) was provided by teachers. The information to be assessed for compliance with the criteria 2 (attendance at the targeted school since the 1st year of ECE), along with criteria 3 (anticipating continued studies in the same school over the following year) and criteria 4 (absence of disorders or risk factors) was provided by the children’s parents. Information related to inclusion criteria 5 (an adequate IQ for their chronological age) was tested using the BADyG-I (*Battery of Differential and General Abilities I*; Yuste and Yuste Peña, 2001).

The initial sample consisted of 44 children who, in addition to complying with the inclusion criteria, presented informed consent forms signed by their parents, authorizing their participation in the study. Six of these children were eliminated from the final sample, since they did not complete all of the activities related to the observation of motor skills and, therefore, had missing data (see the section “Data Analysis”). So, the final sample consisted of 38 children. Of these, 12 participants (31.6%) were male and 26 (68.4%) were female. Their mean age was 5.72 years (*SD* = 0.30). They represented 82.61% of all of the children enrolled in the last year of ECE at the school.

All of the participants were treated in accordance with international ethical principles.

### Natural Setting

Natural setting is one of the requirements for making use of systematic observation (Anguera et al., 2018a, b). In accordance with it, the ECE curriculum (Education and Science Ministry of Spanish Government, 2008; Early Head Start National Resource Center, 2013) has established that the assessment of preschool skills should be carried out in the very educational situations and via direct and systematic observation. Therefore, in our study, the observation sessions intended for assessment of the preschool motor skills took place during the motor development sessions that were carried out regularly in the school (in the motor development classroom) and within their regular scholastic programming.

According to the methodological principles determined by the Spanish government for the learning-teaching process of motor skills in ECE (Health, Social Services and Equality Ministry and Education, Culture and Sports Ministry of Spanish Government, 2017), and, therefore, following the same methodology used by the teachers with their students to prevent any alterations of the regular scholastic context of the study participants, five motor circuits were designed. These circuits were designed to be executed by the participants, in order to observe the specific motor skills of interest for our study. The motor circuit is a methodological proposal in which a set of motor activities are used so that the children could assimilate and improve their motor possibilities through specific and overall work on certain motor patterns, adapted to their level of performance. The activities to be carried out must be explained in advance by the teacher, who acts as a model for the students so that they can visualize the motor patterns to be carried out. The motor circuit must be made up of distinct activities, and its completion requires the execution of a combination of motor skills, which may vary in type: Locomotor skills, Balance, Fine Motor Coordination, etc. (Health, Social Services and Equality Ministry and Education, Culture and Sports Ministry of Spanish Government, 2017).

In accordance with our study’s objective and in line with our initial theoretical framework, the completion of the motor activities forming part of the circuits allowed for the observation of the following specific motor skills: (1) Those belonging to Gross motor skills: (1a) Locomotor skills; (1b) Balance: (i) Dynamic Balance and (ii) Static Balance; (1c) Object Control skills: (i) Propulsive skills and (ii) Receptive skills. (2) Those belonging to Fine motor skills: (2a) Fine Motor Coordination; (2b) Fine Motor Integration.

In order to design the motor activities that allowed for the observation of these specific motor skills, the following aspects were considered: (1) ECE curriculum that specifies the motor skills that should be promoted in children at this age; (2) recommendations and examples of the motor activities proposed by the Health, Social Services and Equality Ministry and Education, Culture and Sports Ministry of Spanish Government (2017) to be carried out in schools in order to improve student motor skills during the period of ECE; (3) existing empirical studies in the scientific literature on the topic that describe activities to be used for the assessment of these childhood motor skills (Franjoine et al., 2010; Grissmer et al., 2010; Pfeiffer et al., 2015; Frick and Möhring, 2016; Gu, 2016; Hestbaek et al., 2017; Oberer et al., 2017; Stein et al., 2017; Cadoret et al., 2018; Chang and Gu, 2018); (4) existing instruments for use in the assessment of motor skills in preschoolers, specifically: the Early Screening Inventory-Revised, 2008 Edition (ESI-R; Meisels et al., 2008); McCarthy Scales of Children’s Abilities (MSCA; McCarthy, 1972); Bruininks–Oseretsky Test of Motor Proficiency-2nd Edition (BOT-2; Bruininks and Bruininks, 2005); Movement Assessment Battery for Children-Second Edition (MABC-2; Henderson et al., 2012); Battelle Developmental Inventory (Newborg et al., 1996); Childhood Neuropsychological Maturity Questionnaire (CUMANIN; Portellano et al., 2000); Pediatric Balance Scale (PBS; Franjoine et al., 2003); and The Beery–Buktenica Developmental Test of Visual-Motor Integration (Beery VMI; Beery et al., 2010).

All of the designed motor activities are playful and fantasy-like. Play is an essential methodological tool of ECE and it should not be separated from a child’s life. Therefore, it is an indispensable tool for the teaching-learning process of children and the observation and analysis of their progress and development (Fasulo et al., 2017; Salcuni et al., 2017; Zosh et al., 2018). But in addition, when play is framed within a world of fantasy, children’s intrinsic motivation, and engagement increase (Garris et al., 2002; Paley, 2005).

The motor activities designed to observe each type of specific motor skill are included in Table 1. Below, each of these is described.

**TABLE 1:** Motor activities designed to observe the distinct specific motor skills.

Principal type of motor skills	Specific motor skills		Motor activities
Gross motor skills	Locomotor skills		Hopping on one leg
			Long jump
	Balance	Dynamic	Walking heel-to-toe
			Jumping in place
		Static	Squatting with arms extended horizontally
			Standing on one leg
	Object Control skills	Propulsive skills	Vertical throwing
			Horizontal throwing
		Receptive skills	Catching a ball
			Catching a bouncing ball
Fine motor skills	Fine Motor Coordination		Tying a pencil
			Touching fingertips
	Fine Motor Integration		Copying shapes
			Copying letters, words, and numbers

(1) Activities to observe Gross motor skills:

(1a) Locomotor skills:

• Hopping on one leg: the child was to hop on one leg down a line, stepping on it without crossing the line. This line was drawn on the ground in a square measuring 1 m per side. Therefore, the child walked on one leg, without stopping, over one of the four sides of the square. On the first two sides, he/she walked with one leg and on the last two sides, with the other.
• Long jump: the participant, situated on a specific point, was to jump forward with both feet together, propelling him/herself forward with his/her arms. The child was to jump as far as possible. Then, he/she was to land on his/her feet, without touching the ground with his/her hands. In the case in which the participant did not comply with these requirements, the jump was considered null and the child was able to repeat the attempt up to a maximum of three times in order to correctly complete the jump.

(1b) Balance:

(i) Dynamic Balance:

• Walking heel-to-toe: the child was to walk down a line with his/her heel next to the toes of the other foot (between the two feet, no space was to be found on the ground). The line that the child was to walk down, without exiting the same and without stopping, was drawn on the ground in a square having 1 m long sides.
• Jumping in place: the participant, situated on a point that is the center of a square measuring 25 cm per side, and looking forward (not at the ground), was to jump up and down 10 times in a row, landing on the same point. While this task was being carried out, an adult counted the jumps, indicating when the participant had completed the 10 jumps so that he/she knew when to stop.

(ii) Static Balance:

• Squatting with arms extended horizontally: the child was to squat over the balls of his/her feet, with feet separated by approximately 30 cm, and with his/her body bent and arms extended horizontally to the sides (that is, their arms extended in the form of a cross). With his/her eyes closed, the child was to remain in this position as long as possible. In the case in which the child remained in this position for less than 5 s, he/she was permitted a second attempt.
• Standing on one leg: the participant, with his/her eyes closed, was to remain standing on one leg on one point. He/she was to remain in this position as long as possible. This exercise was performed on one leg (the leg selected by the participant). In the case in which he/she remained in the position for less than 5 s, the child was permitted a second attempt, with the same leg. Later, the task was carried out using the other leg. Once again, in the case in which the child remains in the position for less than 5 s, he/she was allowed a second chance with this same leg.

(1c) Object Control skills

(i) Propulsive skills:

• Vertical throwing: the child was situated below a hoop measuring 50 cm in diameter and situated 20 cm above his/her head. He/she was to place his/her feet in parallel, slightly separated. From this position, he/she threw a ball (14 cm in diameter) from his/her shoulders upward, sending it through a hoop (once the ball was thrown upward, when falling, it went either inside or outside of the hoop). The child had 4 throws.
• Horizontal throwing: the child was to throw a tennis ball horizontally through a hoop (30 cm in diameter) situated 1.5 m away. He/she had 4 throws with one hand and 4 with the other, beginning with his/her dominant side hand. The ball was thrown from their shoulders, in a straight line and without turning the trunk. The opposite foot (from the throwing hand) was to be placed in front of the other foot.

(ii) Receptive skills:

• Catching a ball: the child was to catch a ball thrown softly by an adult standing in front of him/her at a distance of 1.5 m. The adult had four throws.
• Catching a bouncing ball: the child was to catch a ball after it was bounced from a distance of 0.75 cm. The ball was thrown by an adult standing 1.5 m in front of the child and who threw the ball so that it bounced over a cross that was drawn some 0.75 m from the child, making sure that it bounced directly toward the child. The child was to catch it. The adult had four throws.

(2) Activities to observe the Fine motor skills:

(2a) Fine Motor Coordination:

• Tying a pencil: with a cord measuring 125 cm in length, the child was to form a knot around the pencil so that it was tied by the cord. The child had a maximum of three attempts to successfully complete the task.
• Touching fingertips: the child was to touch his/her thumb to the other fingertips of the same hand, beginning with the pinky finger and going in reverse order (that is from pinky finger, ring finger, middle finger, index finger, middle finger, ring finger, and pinky finger). Once the child completed the exercise with one hand, he/she was to carry it out with the other. The child began with whichever hand he/she wished. He/she had a maximum of three attempts to successfully complete the task.

(2b) Fine Motor Integration:

• Copying shapes: the participant was to copy 6 shapes of distinct complexities (cross, triangle, square, cross made up of an intersection of arrows, rhombus, triangle inscribed inside of another triangle). He/she was given a pencil and sheet of paper with the shapes that were to be copied, having three blank spaces below each shape where he/she could draw his/her copy. The child was not allowed to trace the shape in order to copy it. He/she could use an eraser prior to finishing the shape, but not afterward.
• Copying letters, words and numbers: the participant was to copy 3 letters of distinct complexities (V, H, and T); 3 words of distinct complexities (*TÍO, BOLA*, and *MANO*); and numbers from 1 to 5. The child was given sheets of paper with the letters, words and numbers that he/she was to copy. Under each letter/word/number to be copied, there were three blank spaces where the child could make the copies. The child was not allowed to trace the letter/word/number to be copied. He/she could use an eraser before finishing the letter/word/number, but not afterward.

As previously indicated, the motor circuit, as a methodological proposal, is characterized by different activities that demonstrate distinct motor skills, which may be of different typologies. The circuits intended for ECE students should include a maximum of 4 activities requiring distinct motor skills (Miraflores and Rabadán, 2007). In line with this recommendation, the previously described activities were organized to form five circuits. Each circuit consisted of three distinct activities (see Table 2). (The last activity of circuit 5 –free play– was not considered for this study, and therefore it has not been described).

**TABLE 2:** Motor activities making up each motor circuit.

Motor circuit	Motor activity 1	Motor activity 2	Motor activity 3
1	Hopping on one leg	Tying a pencil	Squatting with arms extended horizontally
2	Catching a ball	Walking heel-to-toe	Long jump
3	Touching fingertips	Vertical throwing	Jumping in place
4	Catching a bouncing ball	Standing on one leg	Copying shapes
5	Horizontal throwing	Copying letters, words and numbers	*Free play*

*Free play = This activity was not considered in this study.*

### Instruments

Given that a mixed methods approach was used, combining observational and selective methodology, distinct types of instruments were used for data collection in this study. First, we present the instruments used in observational data collection and then, those related to the selective methodology. Finally, the data analysis software is indicated.

#### Instruments Used for Observational Data Collection

Systematic observation demands the differentiation and use of distinct types of instruments. Therefore, it is necessary to differentiate between the observation instrument (built *ad hoc* to determine the behaviors of interest, based on the study objective) and recording instruments (used to record and code data).

##### Observation instrument

According to the demands of the observational methodology and taking into account the objective of our study, an *ad hoc* observation instrument was built to observe the motor skills used in each of the activities carried out by the children. Of the distinct types of observational instruments, an instrument that combined a field format and systems of categories was built. A system of exhaustive and mutually exclusive categories was hung from each of the criteria making up the field format. The choice of this type of instrument was justified by the multidimensionality of our observational design.

The instrument was built based on: (a) preliminary recordings of the real object of study; (b) theoretical and empirical studies on motor skills, specifically in childhood (Cameron et al., 2012; Gentier et al., 2013; Pitchford et al., 2016; Hestbaek et al., 2017; Oberer et al., 2017). It was necessary to create distinct versions until reaching the definitive version. The definitive version of the observation instrument is included in the Supplementary Material.

##### Recording instruments

The activity of each participant was recorded using a digital video camera.

Lince (v.1.2.1) (Gabin et al., 2012) free software was used to code observational data from each participant. It may be downloaded from http://lom.observesport.com/.

#### Instrument for Data Collection via Selective Methodology

The BADyG-I (*Battery of Differential and General Abilities I*; Yuste and Yuste Peña, 2001) was used to measure IQ of children and ensure that it was adequate for their chronological age (inclusion criteria 5). It is an instrument that was built, validated, and typified for a Spanish children and with adequate psychometric properties. It is composed by nine subscales. Each subscale has 18 items with five response options (five pictures). The test administrator reads a statement and the child must mark the picture that matches with it. BADyG-I allows to know Verbal, Non-verbal and General Intelligence.

For the evaluation of academic competencies, the PAIB-1 (*Test of basic instrumental aspects: Reading, writing and numeric concepts*; Galve-Manzano et al., 2009) was used in its pencil and paper version. This test allowed for the assessment of literacy and mathematics competencies, obtaining the following: (1) one score referred to Basic Aspects of Reading and Writing; (2) another score referred to Basic Aspects of Mathematics; (3) a total score for all academic competencies (Global Basic Aspects), sum of the 2 previous scores. Therefore, the PAIB-1 permitted the assessment of the most important academic competencies for academic success (Organization for Economic Co-Operation and Development [OECD], 2016), justifying its use as opposed to the use of other standardized instruments that only offer a global score. (Although PAIB-1 also permits the obtaining of other scores, they were not used in this study, given the objective of the same). The reliability of the PAIB-1 has been demonstrated (Galve-Manzano et al., 2009).

#### Data Analysis Software

To control the quality of the observational data, an essential aspect in observational methodology, intra and inter observer reliabilities were calculated using SAS 9.1.3 software (Schlotzhauer and Littell, 1997; SAS Institute Inc, 2004).

To carry out data preprocessing (precisely, to check our dataset for missing data) the ‘VIM’ package (Kowarik and Templ, 2016) of R computing language version 3.6.1 (R Core Team, 2019) was used.

To carry out an analysis that would respond to our study objective (determining which specific preschool motor skills was associated with academic competencies), the R computing language version 3.6.1. (R Core Team, 2019) was used. Specifically, the following packages were used: ‘Stats’ (R Core Team, 2019), ‘QuantPsyc’ (Fletcher, 2012), ‘GGally’ (Schloerke et al., 2020), ‘ggplot2’ (Wickham, 2016), ‘gridExtra’ (Auguie, 2017), ‘lmtest’ (Zeileis and Hothorn, 2002), ‘car’ (Fox and Weisberg, 2019), ‘corrplot’ (Wei and Simko, 2017), and ‘base’ (R Core Team, 2019).

### Procedure

The school management team was informed of the broader research work, of which this study was part, and they agreed to participate in the same. Parents of children enrolled in the 3rd year of the ECE program at this school were also informed. Those wishing to do so (96.4%), authorized the participation of their child in the study. Thus, they signed a consent form, also indicating the required information on their child with regard to sample inclusion criteria 2, 3, and 4 (that is: school attendance at the targeted school since the 1st year of ECE; the intent to continue attending the same school over the following year; absence of disorders or risk factors). All of the children whose parents provided the signed informed consent complied with these inclusion criteria. Later, in order to determine compliance with the sample inclusion criteria 5 (having a suitable IQ for their chronological age), they were assessed using the BADyG-I. Administration of the BADyG-I was collectively carried out in two groups (natural groups of students attending the same class) in two 30-min sessions on non-consecutive days, following the instructions of the test manual. All children presented adequate IQs for their chronological ages. Therefore, they all formed part of the initial study sample.

The first two authors of this manuscript collectively designed each of the motor activities and circuits with the teachers, considering motor skills, educational resources and assessment methodology as determined by the Spanish government for the ECE period. They also considered the spatial conditions of the school’s motor development classroom (where the circuits would be set up) and the temporal organization of each class group (that is, they considered the duration and periodicity of the motor development sessions for each group).

Each class group visited the motor development classroom according to their regular schedule. Each day, one circuit was completed. Before completing the circuit, the teacher explained and demonstrated each of the activities that formed part of a fantasy story to each child. All of the participants performed the activities in the same order (Table 2). Performance of each child on each activity was recorded with a video camera for its subsequent coding and analysis.

Video recordings were imported into the Lince software and were coded using the *ad hoc* observation instrument (available in the Supplementary Material) by two observers who are experts in observational methodology and preschool motor skills (the first two authors of this manuscript). The recorded data were converted into a matrix of codes that was subsequently tested for reliability.

Observational data quality was assured based on two guidelines (Portell et al., 2015a; Anguera et al., 2018a): (a) Qualitative: consensual agreement was used in the first 3 sessions to be codified for each activity (therefore, a total of 42 sessions) by the 2 expert observers; (b) Quantitative: calculating (b1) intra-observer reliability and (b2) inter-observer reliability. (b1) To calculate intra-observer reliability, observer 1, using the unused observation sessions for the calculation of consensual concordance, randomly selected 28 distinct observation sessions (2 sessions of each activity type). (b2) To calculate inter-observer reliability, observer 2 selected another 28 observation sessions, distinct from those used to calculate the consensual concordance and intra-observer reliability, but corresponding to 2 observation sessions for each activity type. Thus, a total of 98 different sessions were used for data quality control. Intra- and inter-observer reliability were calculated through an intra-class correlation coefficient, using SAS 9.1.3 software. In all cases, the intra-class correlation coefficient was ≥0.91. Therefore, the quality of the observational data obtained was excellent.

The following year, when the participants were in the 1st year of primary school education (specifically, at the end of the first quarter), they were administered (in group) the PAIB-1 in order to evaluate their academic competencies. The test was administered in two groups (maintaining children from the same class together for both groups). Test administration was carried out in accordance with the norms indicated in the test manual. So, for each group of children, two sessions were conducted (over non-consecutive days) of 45 and 40 min, respectively. Both the content of the PAIB-1 as well as its format and structure of application were quite similar to the assessment tests carried out by participants on a regular basis in the school.

Test correction was carried out online. Of all of the scores offered by the PAIB-1, in accordance with the study objective, the following three were considered: the score referring to Basic Aspects of Reading and Writing; that referring to Basic Aspects of Mathematics and the score referring to Global Basic Aspects.

### Data Analysis

First, it was necessary to transform the observational data into an appropriate format in order to carry out data analysis. Each category observed in each activity was transformed into a score based on the degree of suitability involved for the execution of said activity (taking the current literature on the topic into consideration: Payne and Isaacs, 2017; Goodway et al., 2019; Haywood and Getchell, 2019). For each participant and activity, the scores corresponding to the observed categories in each activity were added together. Thus, each participant obtained a score for each activity (that is, 14 scores). Later, for each participant, the scores obtained for the two activities referring to each specific motor skills were added together (this relationship between activities and specific motor skills appears in Table 1). In this way, each participant obtained 7 scores, referring to the following specific motor skills: Locomotor skills, Dynamic Balance, Static Balance, Propulsive skills, Receptive skills, Fine Motor Coordination, and Fine Motor Integration.

Data referring to academic competencies did not require transformation.

For both data types, referring to specific motor skills and academic competencies, Multiple Linear Regression analysis was carried out (see the next section). Before carrying out the analysis, the data preprocessing procedure was conducted. We check our dataset for missing data using the R computing language version 3.6.1. More specifically, the aggr() function from the R language package ‘VIM’ was used to visualize the number and proportion of missing values. Schafer (1999) asserted that a missing rate of 5% or less is inconsequential. So, deleting the missing values is a solution when the missing rate is lower than 5% for each variable. We used this solution in our study, given the low missing rate: missing-data rates of 7 variables (4 referring to motor skills and 3 referring to academic competencies) was 0. The rest of the variables (referring to motor skills: Dynamic Balance, Propulsive skills, and Receptive skills) had missing-data rates of 0.046.

#### Multiple Linear Regression Analysis

Multiple Linear Regression (MLR) analysis examines how multiple independent variables are related to a dependent variable (Pedhazur, 1997; Montgomery et al., 2012). The MLR model can accurately reflect correlations between variables, can indicate the degree of fit can improve the effect of the regression equation (Holmes and Rinaman, 2015). In educational research, MLR analysis is commonly used to measure the effects of the explanatory variables on performance (Fariña et al., 2015).

Taking this into account and given our study objective, MLR analysis was used to study the effects of multiple specific preschool motor skills on later childhood academic competencies. MLR modeling was performed using R computing language version 3.6.1. The data analysis procedure for MLR modeling was as follows.

First, the lm() function from the ‘Stats’ core package was used to calculate the MLR models. In line with the aim of our study, 3 MLR models were applied to investigate the effects of multiple preschool motor skills on different childhood academic competencies. More precisely, a model was calculated for each of the 3 dependent variables of interest related to academic competencies: (1) Basic Aspects of Reading and Writing; (2) Basic Aspects of Mathematics, and (3) Global Basic Aspects. Also taking into account our objective, the independent variables included in each of this models were the 7 specific motor skills: Locomotor skills, Dynamic Balance, Static Balance, Propulsive skills, Receptive skills, Fine Motor Coordination, and Fine Motor Integration.

Second, to select the most explanatory variables, we used a mixed stepwise strategy (Osborne, 2017). The mathematical value used to determine the quality of the model was the Akaike Information Criterion (AIC; Akaike, 1973). It helped to make decisions regarding which model was the most appropriate. The model with the lowest AIC value was considered the best at explaining the data. The step() function from the ‘Stats’ package was used for this.

Third, to compare the independent variables included in each model and to determine which one had the strongest relationship with the dependent variable, we generated standardized regression coefficients (β) with the lm.beta() function from the ‘QuantPsyc’ package.

Fourth, analyses were carried out to ensure that there was no violation of the MLR assumption: (a) linearity in the parameters; (b) normal error distribution; (c) homoscedasticity of errors; (d) independence of errors; (e) multicollinearity, and (f) no influential cases (Nimon, 2012; Williams et al., 2013). Different indicators were used to verify these assumptions. They are explained below.

(a) *Linearity*. We examined whether or not the relationship between the independent variables and the dependent variable was linear by looking at: (1) the scatter plot between the dependent variable and each of the independent variables: the points should be distributed around a diagonal line. We used a ggpairs() function from the ‘GGally’ package. (2) The scatter plots between each of the independent variables and the model residuals. If the relationship was linear, the residuals should be distributed randomly around 0 with a constant variability along the *X*-axis. ggplot() function from the ‘ggplot2’ package and grid.arrange() function from the ‘gridExtra’ package were used.
(b) *Normality of distributed errors*. For the MLR analysis, the normality assumption applies to the error distributions (residuals), rather than to predictors and the outcome variables (Williams et al., 2013). This assumption had been verified using: (1) The quantile–quantile plots (Q–Q plot): good alignment to the line should be identified, with no dramatic deviations from it, implying no drastic deviations in error distribution. qqnorm() and qqline functions were used from the ‘Stats’ package. (2) Shapiro–Wilk normality test: It should reveal high *p*-values (*p* > 0.05) to the null hypothesis of the residual being normally distributed. We used the shapiro.test() function from the ‘Stats’ package.
(c) *Homoscedasticity*. Two indicators were used to check homoscedasticity: (1) Scatter plots of residuals: the data points should be distributed above and below zero on the *X*-axis, and above and below zero on the *Y*-axis. When this occurs, it implies a homogeneous distribution of residuals, i.e., the data were homoscedastic. ggplot() function from the ‘ggplot2’ package was used. (2) Breusch–Pagan test: when the Breusch–Pagan test revealed high *p*-values (*p* > 0.05), the null hypothesis of homoscedasticity may be assumed. We used the bptest() function from the ‘lmtest’ package.
(d) *Independent errors*. The Durbin–Watson test was carried out to check for autocorrelation between the investigated variables. This value should lie between the critical cutoff of 1.5 < *d* < 2.5 to assume that there was no first order linear auto-correlation in the multiple linear regression data; that is, the independent errors could be assumed. We used dwt() function from the ‘car’ package.
(e) *Multicollinearity*. Two indicators were used to consider multicollinearity: (1) The correlation matrix between the independent variables: the correlation between the independent variables should be low. When this occurs, it indicates that the independent variables in the model were not correlated and did not provide redundant information about the dependent variable. We used the corrplot() function from the ‘corrplot’ package. (2) The variance inflation factor (VIF): if the VIF values for each independent variable were <10 and even <4 [a more demanding value, defended by other authors such as Hair et al. (2010)], there was no problem with multicollinearity. We used the VIM() function from the ‘car’ package.
(f) *No influential cases*. Cook’s Distance was used to check for no influential cases. Cook’s Distance >1 indicates an influential case (Cook, 1977). Consequently, Cook’s Distance <1 was desirable, suggesting that individual cases were not unduly influencing the model. We used the cooks.distance() function from the ‘base’ package.

## Results

The summary statistics of variables for each MLR model are presented in Table 3. More precisely, the unstandardized beta (*B*), standard error for the unstandardized beta (*SE*), standardized beta or standardized regression coefficients (β), confidence intervals (CI), *t*-statistic values and its *p*-values, multiple *R*-squared (*R*^2^), adjusted *R*-squared (*R*^2^adj) and *F*-statistic are shown.

**TABLE 3:** Summary of statistics of variables of each MLR model calculated.

Dependent variables	Independent variables	*B*	*SE*	β	95% CI	*t*	*p*	Multiple *R*^2^	Adj. *R*^2^	*F*
Basic aspects of reading and writing										
	Intercept	15.000	9.050		-3.372, 33.373	1.657	0.106			
	Coordination	0.165	0.071	0.344	0.021, 0.309	2.334	0.025*	0.239	0.196	5.5**
	Integration	1.373	0.580	0.349	0.196, 2.551	2.367	0.024*			
Basic aspects of mathematics										
	Intercept	–14.107	9.003		-32.366, 4.152	–1.567	0.126	0.227	0.205	10.55**
	Integration	2.009	0.619	0.476	0.754, 3.264	3.248	0.002**			
Global basic aspects model										
	Intercept	–1.785	14.996		-32.228, 28.658	–0.119	0.906			
	Coordination	0.223	0.117	0.267	-0.016, 0.461	1.904	0.065	0.313	0.274	7.981**
	Integration	3.385	0.961	0.493	1.433, 5.337	3.521	0.001**			

p < 0.1, *p < 0.05, **p < 0.01. B, unstandardized beta; SE, standard error for the unstandardized beta; β, standardized beta or standardized regression coefficient; CI, confidence interval; t, t-statistic value; p, p-value of t-statistic; R^2^, multiple R-squared; R^2^adj, adjusted R-squared; F, F-statistic value.

The MLR model that examined the relationship of Basic Aspects of Reading and Writing to motor skills was significant [*F*(2,35) = 5.5, *p* = 0.008]. Basic Aspects of Reading and Writing was positively associated with Coordination (*p* = 0.025) and Integration (*p* = 0.024), explaining 20–24% of its variance (*R*^2^ = 0.239; *R*^2^adj = 0.196). A one-point increase in Coordination score (holding Integration constant) was associated with an increase of 0.165 points in Basic Aspects of Reading and Writing score; a one-point increase in Integration score (holding Coordination constant) was associated with an increase of 1.373 points in Basic Aspects of Reading and Writing score. The magnitude of the β coefficients of Coordination (β = 0.344) and Integration (β = 0.349) was almost equal, thus the weight of each of these specific motor skills in Basic Aspects of Reading and Writing was similar. All of the assumptions of MLR (linearity, normality of distributed errors, homoscedasticity, independent errors, multicollinearity, and no influential case) were met.

In the case of Basic Aspects of Mathematics, the optimal MLR model only included Integration [*F*(1,36) = 10.55, *p* = 0.002]. The association between these two variables was positive. For every additional point on Integration, the Basic Aspects of Mathematics score was 2.009 points higher. Integration explained 21–23% of the variance in Basic Aspects of Mathematics (*R*^2^ = 0.227, *R*^2^adj = 0.205). All of the MLR assumptions were met.

The MLR model testing Global Basic Aspects was significant [*F*(2,35) = 7.981, *p* = 0.001]. Coordination (*p* = 0.065) and Integration (*p* = 0.001) were positively associated with Global Basic Aspects, accounting for 27–31% of its variance (*R*^2^ = 0.313, *R*^2^adj = 0.274). For every additional point in Coordination (holding constant Integration), Global Basic Aspects increased by 0.223 points; for every additional point in Integration (holding constant Coordination), Global Basic Aspects increased by 3.385 points. According to β coefficients, Integration (β = 0.493) had a greater weight on Global Basic Aspects than Coordination (β = 0.267). All of the assumptions of MLR were met.

In summary, the 3 calculated MLR models were significant. In each of these, only one or two independent variables were included, with these always being specific fine motor skills (never specific gross motor skills). The 2 specific fine motor skills (Coordination and Integration) had an association with Basic Aspects of Reading and Writing and Global Basic Aspects. However, only one of them (Integration) revealed an association with Basic Aspects of Mathematics.

## Discussion

The main goal of this study was to determine which specific preschool gross and fine motor skills assessed in Spanish students enrolled in the last year of ECE (5–6 years) were associated with later academic competencies (specifically, in literacy and mathematics and overall) assessed over the following academic year, when students were in their 1st year of Primary Education.

Some of the results obtained are congruent with past findings while others are contradictory. There is no consensus on this topic in the scientific literature and the results are quite disparate. Furthermore, it should be noted that comparison between the results of the diverse studies is complex and should be made with caution, given the distinct sample characteristics from these studies, as well as the variety of motor skills and academic competencies assessed using diverse instruments. In our case, we are unaware of any other studies that have examined the same specific children’s motor skills and academic competencies as those analyzed in our study, using samples of the same age and characteristics, and using the same tasks and assessment tools. All of these variables differ in the studies that have been consulted and this may contribute to the distinct results obtained in each of these (Veldman et al., 2019).

However, and with the previously mentioned caution, we have found our results to be coherent with the literature in terms of the different associations between specific motor skills and academic competencies when this variable (academic competence) was considered globally or specifically, for each main academic domain (literacy and mathematics) (Zhang et al., 2019). Our results are also coherent with other studies in that specific fine motor skills have been found to be more closely linked to academic competencies than specific gross motor skills (Grissmer et al., 2010; Pagani et al., 2010; Pagani and Messier, 2012; Gandhi et al., 2013; Cameron et al., 2016; Pitchford et al., 2016; Zhang et al., 2019). In fact, in our study, specific gross motor skills were not found to be associated with academic competencies, also in accordance with the results of other authors (Grissmer et al., 2010; Pagani et al., 2010; Pagani and Messier, 2012; Gandhi et al., 2013; Pitchford et al., 2016; Macdonald et al., 2020). However, in the literature, certain results contradict this finding: some authors suggest significant associations between gross motor skills and academic competencies both in the literacy and mathematical domains (Son and Meisels, 2006; Pagani et al., 2010; Abdelkarim et al., 2017; de Waal, 2019).

Our results suggest specific associations between the components of fine motor skills (Coordination and Integration) and the academic competencies. Once again, these results are coherent with some past findings, although they disagree with others. And there is a clear lack of consensus in the literature with regard to these relationships, particularly with regard to Coordination. Our results suggest that Coordination is associated with literacy competencies and global academic competency, but not with mathematics competencies. This lack of association between Coordination and mathematical aspects was also supported by Kim et al. (2018). Our results are also congruent with those of other authors who have suggested that Coordination is associated with the literacy domain (Pagani et al., 2010; Dinehart and Manfra, 2013; Manfra et al., 2016; Doyen et al., 2017; Oberer et al., 2018). But unlike our study and that of Kim et al. (2018), these authors have suggested that Coordination is also associated with mathematical competencies. The results of Pitchford et al. (2016) were in partial agreement with those of this series of authors, but they contrasted ours and those of Kim et al. (2018). They found that Coordination was only associated with mathematical aspects, but not with literacy. As for Integration, our results, like those of the literature, revealed associations with both literacy and mathematics competencies (Grissmer et al., 2010; Dinehart and Manfra, 2013; Manfra et al., 2016; Pitchford et al., 2016; Duran et al., 2018; Macdonald et al., 2020).

In summary, our results contribute to the knowledge on the specific relationships existing between the components of gross and fine motor skills in preschoolers and their later academic competencies. However, more research on this topic is necessary, given the disparity of the results found in the literature. Although, as mentioned in the Introduction section, evidence suggests that certain brain structures are responsible for motor and cognitive functions (Diamond, 2000, 2007; Grissmer et al., 2010; Pitchford et al., 2016), our findings suggest that this does not guarantee that all specific motor skills scores are significantly associated with all academic competencies score (Chagas et al., 2016).

On the other hand, as shown in all cases, the percentage of variability in academic competencies that is explained by the different specific motor skills varied from *R*^2^adj = 0.196 to *R*^2^adj = 0.274. In the humanities and social sciences, these *R*^2^adj values lie within an acceptable range, with these values being quite typical (and even other lower ones) in many education studies, some of which also focus on the explanation of academic performance (Oliveira et al., 2017; Pires et al., 2017; Cueli et al., 2019; Morales-Rodríguez et al., 2019; Schorr, 2019; Xiao et al., 2019; Tinajero et al., 2020). Social phenomena are complex and multidimensional, so it is not expected that all relevant variables indicating the subject’s behavior will be included. Therefore, it is very difficult to explain a very large amount of variation (Neter et al., 2012; Xiao et al., 2019). Also, it should be highlighted that even if the *R*^2^adj values were considered to be low, the low *p*-values suggest that motor skills included in the models were significantly correlated with academic competencies, meaning that important conclusions could still be drawn from the models (Neter et al., 2012). Ultimately, in explaining human behavior, even small values of *R*^2^adj can be quite meaningful. They can certainly be used to better understand the phenomenon under study (Abelson, 1985; Moksony, 1999). But, of course, it would be interesting for future studies to include other variables that affect the academic competencies, in addition to those studied here, such as additional personal factors of students (birth weight, gestation period, children’s emotions, behavior problems, cognitive dimensions, etc.), family characteristics (educational style of parents, socioeconomic status, etc.) and aspects of the school system (teacher expectations of students, pupil-teacher relationships, etc.) (Pires et al., 2017; Zhang et al., 2019). This would permit an increased understanding of the topic and the design of optimal interventions to improve students’ academic competencies.

The results of our study have numerous educational implications. Therefore, we believe that it is necessary to clarify the following point: Although our results do not reveal significantly positive associations between gross motor skills and academic competencies, negative associations were not found. So, our results do not suggest that gross motor skills should be discarded by ECE teachers. The goal of this educational period is to contribute to the child’s overall development, and since numerous works have affirmed that gross motor skills promote childhood social competencies development (Goodway et al., 2019; Haywood and Getchell, 2019), their inclusion in preschooler educational practices is more than justified.

Our results highlight the importance of fine motor skills in ECE in order to strengthen subsequent academic achievement. However, frequently, the reality of the classroom situation is quite different. Motor skills, in general, are not highly valued, and therefore, they may not be promoted to the extent that is truly necessary. This appears to be due mainly to the common misconception that children develop motor skills in a natural manner, during their maturation process. However, motor skills do not emerge naturally. They must be learned, practiced, and reinforced (Logan et al., 2012; Pic et al., 2018, 2020). But, this is often forgotten, given the pressure existing in the lower education levels toward instrumental learning. As a result, many childcare settings focus exclusively on academic content, making young children’s opportunities to advance motor skills in these settings increasingly limited (Cameron et al., 2016; Osborne et al., 2016; Macdonald et al., 2020).

Research on motor skills suggests that these skills are fundamental for early learning and that they are quite malleable during this early childhood period. In fact, preschool years (3–5 years of age) have been called the “golden age” for motor skills development and learning, suggesting possible windows of intervention (Shenouda et al., 2011). In Spain (and also in most of the other European countries), almost all children of these ages attend ECE, even though it is not a mandatory school phase (European Commission/EACEA/Eurydice, 2019; Spanish Ministry of Education and Professional Training, 2019). Therefore, it is an ideal opportunity for all children, regardless of their socioeconomic background, to develop and improve their motor skills. Furthermore, early education teachers form a part of the children’s micro context, monitoring these children for longer periods of time and gaining the trust of their families. This makes them the ideal candidate to promote motor skills and identify early motor problems, and to implement planned programs that prevent or mitigate them (Cueto et al., 2017; Tsangaridou, 2017). These planned movement activities should form a routine part of the preschool curriculum and should be based on play, more specifically, guided play (in which an adult selects or arranges a context for learning but the children direct the play; although the adult may provide scaffolding and guidance), since this is the most natural way of learning and developing in children (Weisberg and Zosh, 2018; Yu et al., 2018; Zosh et al., 2018).

However, it is necessary for ECE teachers to increase play-based opportunities to ensure the development of children’s motor skills. Environment-initiated practice opportunities greatly influence both the rate and direction of motor development (Ward et al., 2019). Early motor stimulation before the child engages in formal learning helps to create neuronal networks that may facilitate school readiness and academic knowledge acquisition (Thomas et al., 2019).

But in order to plan successful opportunities to promote the development of children’s motor skills, adequate prior assessment is necessary. ECE teachers have declared that they lack the skills and knowledge necessary to assess children’s motor skills and therefore, may be unable to engage in appropriate motor practices for young children (Gehris et al., 2015; Cueto et al., 2017). This may be one of the reasons why motor skills are not being promoted in ECE to the extent that they should be. The ECE curriculum in Spain and other European countries declares that action and movement are essential principles that must be acquired during this educational period (European Commission/EACEA/Eurydice, 2019). So, ECE teachers are required to assess motor skills. However, the literature indicates that many motor skill assessments carried out by the ECE teachers are based on impressions and personal subjectivity, given a lack of training on the same. Teachers indicate that their limited knowledge, especially of assessment techniques, significantly limits their use of measurement tools which may provide objective and precise information (Cueto et al., 2017). In fact, the literature indicates that only 54.7% of the ECE teachers are capable of correctly assessing their students’ motor skills. Of those committing assessment errors (improperly assessing their students’ motor skills), 91.5% overestimate their students’ abilities. And even more alarmingly, 13.27% of the teachers assess their students’ motor skills as being normal when in fact, they suffer from deficits (Cueto et al., 2017). This may have major consequences on the student’s development and learning over the short, medium and long term, since they may be denying the possibility of necessary intervention that would improve their motor skills, thus, limiting their later academic achievement. This lack of preparation by ECE teachers on suitable assessment and intervention of motor skills is not limited to Spain, but is a global problem, as reported over and over in the international scientific literature (Robinson et al., 2012; Gehris et al., 2015; Battaglia et al., 2019).

So, one of the clear highlights of our study is that it offers a tool (and even better, a free tool) that permits to objectively assess motor skills in the children’s natural educational context (see the *ad hoc* observation instrument, available in the Supplementary Material). So, our study responds to the current need to construct practical tools that permit the assessment of motor skills by ECE teachers (Cueto et al., 2017); and more specifically, the need to increase the limited number of observational tools currently available to teachers in order to identify children having motor problems (Figueroa and An, 2017). Based on these assessments, it would be possible to design and implement teaching practices and interventions that are suited to the specific needs of the students. The built tool would also be useful to assess progress and evaluate the efficacy of these practices and interventions, permitting their modification and thus, offering an evidence-based practice. All of this would clearly improve the early motor skills and offer the possibility of improved future academic achievement for the children.

Another highlight of our work is the fine-grained assessment of the distinct components of motor skills. We have assessed all of the specific preschool motor skills, both gross and fine, identified in the reviewed literature, thereby addressing the multidimensional nature of motor skills and their specific relationships with academic competencies. Many authors criticize the fact that many studies report motor outcomes and only include one overall motor skill or, when including various measures, all of them come from one instrument that is used to assess the holistic development of the children instead of specific components of motor child development (Kim and Cameron, 2016; Macdonald et al., 2018). Therefore, our study extends beyond the limited view of motor skills that has been commonly presented in empirical scientific studies.

The same occurs with regard to academic competencies. Studies reporting academic outcomes often offer only one overall academic performance score (i.e., a combination of literacy and mathematics) (Macdonald et al., 2018). However, in our study we have adopted a more multidimensional approach, considering not only an overall academic competency score, but also analyzing the literacy and mathematics competencies separately. And we have used a standardized instrument to do so, therefore offering a more objective, reliable and valid assessment as compared to the use of only qualifications provided by teachers (Marzano, 2000; Organization for Economic Co-Operation and Development [OECD], 2012; Castejón et al., 2016; Meissel et al., 2017).

Thus, all of the aspects considered in our study respond to suggestions made by various authors (Son and Meisels, 2006; Kim and Cameron, 2016; Macdonald et al., 2018) who have proposed that future studies employ in-depth assessment of motor skills and academic achievement, including diverse aspects of motor skills and academic competencies, in order to better determine which specific aspects of motor skills are associated with which specific aspects of academic achievement. However, to extend upon our study, future works should consider an even broader perspective on these academic competencies than that considered here, including more specific scores on diverse aspects of the linguistic domain (e.g., vocabulary and spelling) and mathematics (operations, measurement, etc.). This would provide even more knowledge on the specific relationships between motor skills and academic competencies, offering more support for the inclusion of certain types of activities at school.

We also believe that future works should continue to examine not only which motor and academic aspects are specifically related, but also how and why. The use of other data analysis techniques, in addition to educational neuroscience studies may provide additional knowledge to optimize learning practice (Coch, 2018; Thomas et al., 2019).

So, in our study, we highlight the use of two motor activities to assess each of the specific motor skills, as opposed to only one, as is the case in most studies (Tomac et al., 2012). This is in line with recommendations of other authors, although it implies a major effort (Cameron et al., 2016). Assessing each specific motor skill in two activities provides additional information on the children’s level of each specific gross and fine motor skill. However, it is clear that in the analyses of this study, we have not separately considered the motor performance carried out in each of these activities. Future studies should examine whether or not the level of execution differs in each of the two activities in which each specific gross and fine motor skill was assessed. Studying the different demand characteristics across the spectrum of motor activities could help illuminate why and when a particular task and a particular skill contribute to various educational outcomes (Cameron et al., 2016). It may also assist in instruction, assessment, and intervention programs.

It is important to indicate that in this study, we have attempted to describe each of these activities in detail, in response to criticisms of other works that failed to specify the tasks used for motor skills assessment (typically only mentioning the tasks, without providing any additional information). Using a certain activity to assess the same motor skill may result in distinct results. Therefore, failing to offer a detailed explanation of the tasks used may hinder the comparison of results of different studies (Veldman et al., 2019).

In addition, the descriptions of activities and circuits that we have presented in this study may also help the professional practice of ECE teachers as it offers examples of specific motor activities that can be applied to children (specifically, 5–6 year-olds), designed with consideration to the ECE curriculum and recommendations of the Spanish Health, Social Services and Equality Ministry and the Spanish Education, Culture and Sports Ministry. Often, teachers complain of a lack of resources and materials available to implement practices that improve preschool motor skills, as well as their lack of training and time to design the same (Robinson et al., 2012). Therefore, this work may be useful.

On the other hand, this study has examined the motor skills of children during a changing moment, which, despite being of special importance for their development and learning, has barely been considered in research. In general, motor skills tend to be studied at a very young age, based on a clinical perspective, focusing on dysfunctions or inefficient movement behavior, or at later ages, with regard to athletic skills (Altunsöz, 2015). So, despite its importance, research on motor skills in preschool children is scarce and quite fragmentary (Cools et al., 2009). Our study has attempted to contribute to the elimination of this gap. Many authors have defended the potential usefulness of assessing preschool motor skills in a normative sample and in an educational context, as was done in this study (Hyson and Douglass, 2019).

Our results reveal associations between specific fine motor skills of preschoolers and their academic competencies one school year later, in the 1st year of Primary Education. It would be interesting to carry out a follow up study to determine if the relationship between specific preschooler motor skills and academic competencies differ during Primary Education grades and beyond.

Of course, in the future it may be necessary to also increase sample size, given its small size is one of the main limitations of this study. However, difficulties in doing arise when working with minors (Fargas-Malet et al., 2010; Elwick et al., 2014): (1) it is necessary to rely on the cooperation of different “gatekeepers,” such as school staff and parents. At times, the school staff is so overworked that, even though the importance of the study is recognized, they do not collaborate. With regard to parents, it is not unusual overprotective attitudes toward their children and consequently, the non-authorization for participation of their children in studies. (2) The evolving characteristics of the children, such as their great degree of distraction even when playing, high fluctuation of motivation and rapid fatigue (Zosh et al., 2018), requiring greater time and effort in data collection.

Other issue that also justifies the smaller sample size is the use of observational methodology. This methodology is distinguished by its intensive nature as compared to the extensive nature of other methodologies; i.e., there is a greater interest in obtaining a large quantity of detailed information on the natural behavior of a small number of participants than in the representativeness with respect to a larger population (Anguera, 2003). In addition, in order to capture the richness of information on participant behavior, this methodology demands the building of a reliable and precise *ad hoc* observation instrument (in this case, it is available in the Supplementary Material). This implies arduous and detailed work. Other characteristics of the observational methodology (such as the need to rely on expert observers or to devote time to their training, and the need to carry out rigorous quality control of the data) require much time, effort and cost for the researchers (Portell et al., 2015b; Bardid et al., 2019; Maddox, 2019).

All of this serves to explain and justify why observational studies, and hence, our study, are carried out using a smaller, accessibility-based sample. In fact, the high cost involved in observational studies, even when recognizing their greater suitability in examining child development and learning, has led to a preferred use of other, less costly methodologies (Maddox, 2019). Little value has been placed on the extra effort made in studies involving the direct behavioral observation and the increased effort required when observing the behavior of young participants (Patterson, 2008). Furthermore, it should be noted that observational studies are often “punished,” considering them to be undervalued, based on the predominantly scientific-based perspective of the experimental paradigms (Rozin, 2009). So, we advocate a correct and just assessment of observational studies, defending their use, despite the effort that they imply in many aspects, since this methodology is the most appropriate and possibly even the only potential option for the study of spontaneous childhood behavior in an educational setting (Anguera, 2001; Escolano-Pérez et al., 2017, 2019a,b; Bardid et al., 2019; Vitiello et al., 2019). Systematic observation provides rich and contextualized information that cannot be obtained from other methodologies, making it possible to gather relevant data to describe, explain, and understand fundamental aspects of children’s development and learning (Federici et al., 2017; Otsuka and Jay, 2017). So, more observational studies are clearly needed in order to better understand childhood development and learning and thereby implement more effective early educational practices.

## Conclusion

The mixed methods approach used has allowed us to know that only the fine motor skills, and not the gross ones, of preschool children (5 to 6-year-olds) are associated with their academic competencies 1 year later, when the students were in their 1st year of Primary Education. These results highlight the importance of preschool fine motor skills in order to strengthen subsequent academic achievement. Given fine motor skills are quite malleable during this early childhood period, ECE teachers have to increase play-based opportunities to ensure and promote the development of children’s fine motor skills. This early educational practices will encourage future academic achievement.

## Data Availability Statement

The raw data supporting the conclusions of this article will be made available by the authors, without undue reservation, to any qualified researcher.

## Ethics Statement

This study was part of a broader research study that was evaluated and approved by the Research Unit of the University of Zaragoza. Research was also approved by the school management team. In accordance with Organic Law 15/1999 of December on Protection of Personal Data (1999, BOE no. 298 of December 14), all parents of the participants signed the informed consent authorizing the participation of their children in the study and their being recorded. In addition, and following the guidelines of the aforementioned law, observers signed the confidentiality agreement. No special ethical approval was required for this research since the Spanish public education system and national regulations do not require such approval. Each participant received a small reward (two chocolates) in gratitude for their participation.

## Author Contributions

EE-P was involved in conceptual and methodological structure, literature review, collecting data, systematic observation, manuscript drafting, data analysis, results, and discussion. MH-N was involved in data collection and systematic observation. JL was involved in methodological structure and data analysis. All authors contributed to revising the manuscript and provided final approval of the version to be published.

## Conflict of Interest

The authors declare that the research was conducted in the absence of any commercial or financial relationships that could be construed as a potential conflict of interest.
