# Supplementary material for: Association Between Preschoolers’ Specific Fine (But Not Gross) Motor Skills and Later Academic Competencies: Educational Implications
Source: Front Psychol. 2020 Jun 5;11:1044. doi: 10.3389/fpsyg.2020.01044 (PMC7289974; doi:10.3389/fpsyg.2020.01044)
Supplement: Supplementary file 1 [file Data_Sheet_1.pdf]

| *Criterion* | *Criterion description* | *Category systems* | *Category description* | *Category code* |
|---|---|---|---|---|
| Participant | Child carrying out the motor activity. | Participant 1 | Participant 1 carries out the motor activity. | P1 |
| | | Participant 2 | Participant 2 carries out the motor activity. | P2 |
| | | ... | The participant... carries out the motor activity. | Pn |
| Motor activity | Activity performed by the participant. | Hopping on one leg | Activity in which the participant should hop on one leg down a line, stepping on it and without crossing it. | Apc |
| | | Long jump | Activity in which the participant, situated on a specific point, jumps forward with both feet together, propelling him/herself with his/her arms. The participant should land on his/her feet, not touching the ground with his/her hands. | Sl |
| | | Walking heel-to-toe | Activity in which the participant should walk down a line with his/her heel next to the toes of the other foot (there should be no space left on the ground between the two feet). | Apt |
| | | Jumping in place | Activity in which the participant, situated on a point that is the center of a square measuring 25 cm per side, and looking forward, should jump up and down ten times in a row, landing on the same point where he/she began. | Ss |
| | | Squatting with arms extended horizontally | Activity in which the participant, with eyes closed, should remain in a squatting position over the balls of his/her feet, which should be separated by approximately 30 cm, with his/her body bent and arms extended horizontally to the sides (that is, extended in the form of a cross). | C |
| | | Standing on one leg | Activity in which the participant, with eyes closed, should remain standing on one leg, on the same point, first standing on one leg and then changing to the other. | Ppc |
| | | Vertical throwing | Activity in which the participant, with feet parallel and slightly separated, should throw a ball up in the air and through a hoop that is situated above him/her. | Lv |
| | | Horizontal throwing | Activity in which the participant should throw a tennis ball horizontally so that it passes through a hoop that is situated at a distance of 1.5 m away from them. | Lh |
| | | Catching a ball | Activity in which the participant should catch a ball that is thrown by an adult from a distance of 1.5 m. | Rp |
| | | Catching a bouncing ball | Activity in which the participant should catch a ball that is bounced on the ground at a distance of 0.75 m. | Rpb |
| | | Tying a pencil | Activity in which the participant should tie a knot around a pencil so that the pencil is tied with the cord. | Al |
| | | Touching fingertips | Activity in which the participant should use their thumb to touch the fingertips of the other fingers of the same hand, one at a time, first touching the pinky finger, and continuing in reverse order. | Td |
| | | Coping shapes | Activity in which the participant should copy 6 shapes of distinct complexities. | Cf |
| | | Copying letters, words and numbers | Activity in which the participant should copy 3 letters of distinct complexities; 3 words of distinct complexities; and numbers from 1 to 5. | Cl |
| Specific motor skill | Specific motor skill carried out by the participant. | Locomotor skills | Actions of large muscle and postural groups that involve the coordination of the entire body, permitting the body's transfer from one point in space to another, through body movement. | Ls |
| | | Dynamic Balance | The ability to hold a position during activities requiring movement, such as walking. It is obtained when the body's stability is maintained during movement performance. | Db |
| | | Static Balance | The ability to hold a position during stationary tasks such as standing or sitting. | Sb |
| | | Propulsive skills | They involve sending an object away from the body. | Ps |
| | | Receptive skills | They involve receiving an object. | Rs |
| | | Fine Motor Coordination | It refers to small muscle movements but not to the integration of these muscle movements with other input, such as visual-spatial information, from the environment. | Cmf |
| | | Fine Motor Integration | It involves the organization of small muscle movements in the hand and fingers with the processing of visual stimuli. Visual information from the environment must be processed and integrated with fine motor movements. | It |

| | | | | |
|---|---|---|---|---|
| Side of a square | Side of the square on which the participant carrying out the activity is situated. This applies in the following activities: *Hopping on one leg* and *Walking heel-to-toe*. | Side 1 | The participant is carrying out the activity on side 1 of the square. | L1 |
| | | Side 2 | The participant is carrying out the activity on side 2 of the square. | L2 |
| | | Side 3 | The participant is carrying out the activity on side 3 of the square. | L3 |
| | | Side 4 | The participant is carrying out the activity on side 4 of the square. | L4 |
| Trajectory | Route or means adopted by the participant's movement when carrying out the activity; that is, the line described by the participant when he/she moves through the space when carrying out the activity.<br>It applies to the following activities: *Hopping on one leg* and *Walking heel-to-toe*. | Rectilinear | The participant's movement adopts a straight line. Specifically, this implies that when landing from the jumps (if carrying out the *Hopping on one leg* activity*)* or in his/her steps (if carrying out the *Walking heel-to-toe* activity), the participant steps on the central line (blue) with the entire foot or part of it. | TR |
| | | Oscillating | The participant's movement is fluctuating, so that in at least one of the landings from their jumps (if carrying out the *Hopping on one leg* activity) or in one of their steps (if carrying out the *Walking heel-to-toe* activity), the participant does not cross the central line (blue) but instead, steps either to the right (space marked out by the blue stripe and the red one) or to the left (space marked by the blue stripe and green one). That is, in at least one of the landings from their jumps, or in one of their steps, the participant does not step on the central blue line, but on the space that is marked out by the two lateral lines (red and green). | TO |
| | | Erratic | The participant's movement is incorrect, so that, in at least one of the landings from their jumps (if carrying out the *Hopping on one leg* activity) or in one of their steps (if carrying out the *Walking heel-to-toe* activity), the participant does not step on the central line (blue) or within the space marked out by the two lateral lines (red and green). He/she rather, steps outside this space. That is, the participant steps either very far to their right (beyond the lateral red line) or very far to their left (beyond the lateral green line). | TE |
| Cadence | Regularity of the series of movements carried out by the participant. It applies in the following activities: *Hopping on one leg* and *Walking heel-to-toe.* | Rhythmic | The movement is carried out at a constant speed, without interruptions, stops or changes in speed. | CR |
| | | Arrhythmic | The movement is carried out at a varying speed, with interruptions, stops and/or changes in speed. | CA |
| Extremity | Upper or lower limb (depending on the activity at hand) with which the participant carries out the movement. It applies to the following activity: *Hopping on one leg, Horizontal throwing, Touching fingertips* and *Standing on one leg*. | Right | The (upper or lower) right limb is used by the participant to carry out the movement. | ED |
| | | Left | The (upper or lower) left limb is used by the participant to carry out the movement. | EIz |
| Arm position | Posture in which the arms are positioned while the participant performs the action. It applies to the following activities: *Hopping on one leg, Squatting with arms extended horizontally, Long jump* (to each of the jump phases), *Horizontal throwing* and *Vertical throwing.* | Backwards | The arms are placed behind the participant's shoulders while he/she performs the action (which may be a throw from the back to the front or another action with the arm). | PbA |
| | | Forwards | The arms are placed in front of the participant's shoulders while he/she performs the action, without having been, at any time, behind them during the course of the action. | PbD |
| | | Across the body | The arms are extended outwards, following the directionality of the participant's body while he/she performs the action. | PbC |
| | | In the form of a cross with arms extended | Upon performing the activity, the participant keeps his/her arms extended horizontally in the form of a cross. | PbCzE |
| | | In the form of a cross with arms bent | Upon performing the activity, the participant keeps his/her arms horizontally bent in the form of a cross. | PbCzFl |
| | | Others | Upon performing the activity, the participant keeps his/her arms in a position that differs from those mentioned above. | PbO |
| Jump phase | One of the three phases that may be distinguished in the jump, that is, one of the three moments that can be distinguished in the movement produced by the action of one or both legs, through which the participant's body leaves the ground as a support surface, later returning to it. This applies to the *Long jump* activity. | Impulse | Jump phase where the trajectory to be followed by the participant is determined. It requires a bending, followed by a quick extension of the lower trunk. | FsIm |
| | | Flight | Jump phase in which the participant loses contact with the surface. It implies a horizontal displacement across the air. | FsV |
| | | Landing | Jump phase in which the participant comes into contact with the ground once again. In this phase, the accumulated energy is absorbed or transformed. | FsR |

| | | | | |
|---|---|---|---|---|
| Leg position | Posture adopted by the participant's legs when carrying out the following activities: *Long jump* (in each of the phases of the jump), *Catching a ball* and *Catching a bouncing ball.* | Knees bent | While performing the activity, the participant keeps his/her knees bent, so that his/her abductor muscles and calves are close together. | PRfl |
| | | Knees not bent | While performing the activity, the participant keeps his/her knees extended, without bending them. | PRsfl |
| Distance to the ground | Space between the ground and the participant's feet. This applies to the following activities: *Long jump*, *Squatting with arms extended horizontally* and *Jumping in place*. In the *Long jump* activity, it is applicable to each of the jump phases. | Feet on the floor | The balls of the participant's feet are kept fully on the ground. (In the *Jumping in place* activity, this only applies to the landing of the jump phase). | DsPs |
| | | Heels lifted | Only the balls of the participant's feet touch the ground, with their heels being lifted up from the ground. | DsTe |
| | | Feet in the air | The participant's feet are in the air, not touching the ground with any part of them. This category is not applicable to the *Squatting with arms extended horizontally* activity. | DsPa |
| Centimeters | Space, distance measured in cm from the point where the propelling takes place, as done by the participant when jumping, to the point where the landing of said jump occurs. This only applies to the *Long jump* activity. | Quartile 1 distance | The participant has jumped a distance that is situated between 0 and 25% from the lowest distances jumped by the study participants. | C1 |
| | | Quartile 2 distance | The participant has jumped a distance that is situated between 26% and 50% from the lowest distances jumped by the study participants. | C2 |
| | | Quartile 3 distance | The participant has jumped a distance that is situated between 51% and 75% from the greatest distances jumped by the study participants. | C3 |
| | | Quartile 4 distance | The participant has jumped a distance that is situated between 76% and 100% from the greatest distances jumped by the study participants. | C4 |
| Base of support | Space, distance between the participant's feet. This applies to the following activities: *Long jump* (to each of the jump phases), *Jumping in place* (to the landing of the jump), *Horizontal throwing* and *Vertical throwing.* | Feet together | The participant's feet touch one another. | BsPj |
| | | Feet separated | The participant's feet are separated by a distance of less than 15 cm. | BsPs |
| | | Feet widely separated | The participant's feet are separated by a distance of more than 15 cm. | BsPms |
| Type of landing | Means in which the landing of the jump takes place in the *Long jump* activity. This only applies to this activity. | Without bouncing | During the landing phase of the jump in the *Long jump* activity, the participant keeps his/her feet immobile with respect to the first position in which he/she landed. It is considered a correct jump. | TcSr |
| | | With a bounce | During the landing phase of the jump in the *Long jump* activity, the participant moves his/her feet with respect to the first position in which he/she landed. It is considered an incorrect jump. | TcCr |
| Precision of the jump | In the *Jumping in place* activity, adjustment between the position of the feet in the jump landing phase and the square that is painted on the ground (participant location at the start of the activity). That is, this category assesses if the participant, during each jump, lands in the same area where he/she began the task. | The 2 feet within the square | During the landing phase of the jump, the participant lands with the 2 feet within the square, without stepping on any of its lines. | Ps2Dt |
| | | At least one foot steps on a line of the square | During the landing phase of the jump, the participant lands stepping on at least one of the lines of the square. | Ps1Dt |
| | | Outside of the square | During the landing phase of the jump, the participant lands with at least one foot completely outside of the square. | PsF |
| Trunk position | Position adopted by the torso. It applies to the following activities: *Squatting with arms extended horizontally*, *Horizontal throwing*, *Vertical throwing*, *Catching a ball* and *Catching a bouncing ball.* | Upright | The participant's torso is positioned vertically with respect to the ground, that is, their torso is at a 90º angle to the ground. | PtE |
| | | Inclined | The participant's torso is not vertical with respect to the ground, that is, the participant's torso forms an acute or obtuse angle with the ground. | PtI |
| Time | Seconds in which the participant carries out the activity. It applies to the following activities: *Squatting with arms extended horizontally* and *Standing on one leg.* | Quartile 1 time | The time that the participant spends performing the activity falls within quartile 1 of the times used by all of the participants. | T1 |
| | | Quartile 2 time | The time that the participant spends performing the activity falls within quartile 2 of the times used by all of the participants. | T2 |
| | | Quartile 3 time | The time that the participant spends performing the activity falls within quartile 3 of the times used by all of the participants. | T3 |
| | | Quartile 4 time | The time that the participant spends performing the activity falls within quartile 4 of the times used by all of the participants. | T4 |
| Direction of | Position of the leg that is not standing on the ground. | Backwards | The leg that is in the air is positioned behind the shoulders (regardless of the angle formed by the bending | DplA |

| | | | | |
|---|---|---|---|---|
| lifted leg | This only applies to the *Standing on one leg* activity. | | of the knee). | |
| | | Laterally | The participant lifts the leg in the air to one side or another (right or left), in the direction of the shoulder (regardless of the angle formed by the bending of the knee). | DplL |
| | | Other | The participant puts the lifted leg in any position other than the previous ones. | DpñO |
| Angle of the lifted leg | Angle of the leg that is not standing on the ground. This only applies to the *Standing on one leg* activity. | Acute angle | The leg that is not standing on the ground is bent at an angle of less than 90º. | AplA |
| | | Right angle | The leg that is not standing on the ground is bent at an angle of 90º. | AplR |
| | | Obtuse angle | The leg that is not standing on the ground is bent at an angle of over 90º. | AplO |
| Tying | Movement made with a cord to attempt to tie a knot around a pencil so that the pencil is supported. This applies to the *Tying a pencil* activity. | Pencil on the cord | The participant places a pencil on the cord but does not perform any other action. | Alec |
| | | Around the pencil | The participant puts a pencil on the cord and then places the cord around the pencil, that is, he/she surrounds the pencil with the cord but does not cross the ends of the cord. | Aalr |
| | | Crossing the ends of the cord | The participant puts a pencil on the cord and then crosses the ends of the cord (that is, passes the end of the left side to the right and that of the right side to the left, ending up with one end of the cord over the other), but without tying them (without tying or joining them). | Acex |
| | | Tying the cord | The participant puts a pencil on the cord and then crosses the ends of the cord (that is, passes the end of the left side to the right and that of the right side to the left, ending up with one end of the cord over the other) and ties them together (tying them together, joining them) around the pencil. | Acrz |
| | | Stretching the ends | The participant puts a pencil on the cord and then crosses the ends of the cord (that is, passes the end of the left side to the right and that of the right side to the left, ending up with one end of the cord over the other) and twists them together (tying them together, joining them) around the pencil. In addition, he/she pulls both ends of the cord, tightening it to the pencil. This suggests that the participant is capable of successfully tying the knot (since upon lifting the cord in the air, the pencil will not fall, as it is tied). | Aext |
| Finger | Finger touched by the participant with his/her thumb. This applies to the *Touching fingertips* activity. | Pinky finger | Three-phalange finger, located on the external side of the hand. | DM |
| | | Ring finger | Three-phalange finger, located next to the pinky finger. | DAn |
| | | Middle finger | Longest three-phalange finger, located in the center of the hand. | DCo |
| | | Index finger | Three-phalange finger, located next to the thumb, between this and the middle finger. | DIn |
| Direction | Direction or order in which each finger is touched within the touching sequence. This only applies to the *Touching fingertips* activity. | Direct | Starting with the pinky finger, the participant touches the other fingers until reaching the index finger. | Sdi |
| | | Reverse | Starting with the index finger, the participant touches the other fingers until ending with the pinky finger. | Sinv |
| Part of the finger | Part of the finger that is touched by the thumb. This only applies to the *Touching fingertips* activity. | Fingertip | The participant touches one of his/her fingertips with his/her thumb. That is, the participant’s thumb touches a finger on its upper part, on the opposite side of the nail. | PdYe |
| | | Other | The participant touches another finger with his/her thumb on any of its parts other than the fingertip. | PdOt |
| Way of catching the ball | Way in which the participant catches the ball that has been thrown, using or not using his/her body to help. This only applies to the *Catching a ball* and *Catching a bouncing ball* activities. | With both hands | The participant catches the ball with both arms and hands held forward and separated from his/her body, holding it between his/her hands. | Rp2m |
| | | Supporting on body | The participant uses his/her body to catch the ball, supporting the ball with their body. | RAac |
| | | Not catching | The participant does not catch the ball so that it falls to the ground, even if it was previously touched with the hands or another body part, or even when it was previously caught for a brief period of time. (That is, this category is coded regardless of whether the participant momentarily trapped the ball but it immediately fell out of his/her hands). | RpNr |
| Hand position | Posture adopted by the participant’s hands when catching the ball. This only applies to the *Catching a ball* and *Catching a bouncing ball* activities. | Together | The participant catches the ball with both hands touching each other, with their palms facing upward. | PminJ |
| | | Separate | The participant catches the ball with his/her hands separated, situated one on each side of the ball. | PmS |
| Height of the catch | Vertical distance at which the ball is recovered with respect to the ground. This only applies to the *Catching a ball* and *Catching a bouncing ball* activities. | Chest | The participant catches the ball at chest height. | ArP |
| | | Neck head | The participant catches the ball at face or neck height. | ArCc |
| | | Abdomen | The participant catches the ball at abdomen height. | ArAb |

| | | | | |
|---|---|---|---|---|
| | | Thighs | The participant catches the ball at thighs height. | ArM |
| | | Knee | The participant catches the ball at knees height. | ArRd |
| | | Under the knee | The participant catches the ball when it is below his/her knees. | ArDrd |
| Attempt | A participant's attempt to perform the task. This applies to the *Long jump, Squatting with arms extended horizontally, Standing on one leg, Tying a pencil, Touching fingertips*, *Copying shapes* and *Copying letters, words and numbers* activities. | 1 | The participant performs the task for the first time. | I1 |
| | | 2 | The participant performs the task for the second time. | I2 |
| | | 3 | The participant performs the task for the third time. | I3 |
| | | +3 | The participant performs the task for the fourth time, or more. | Im3 |
| Passing through | It indicates whether or not the ball passes through the hoop. This applies to the *Vertical throwing* and *Horizontal throwing* activities. | Transfer | The ball passes through the hoop. | AtEc |
| | | Touch | The ball touches the hoop but does not pass through it. | AtTn |
| | | Diverted | The ball is diverted and does not pass through or touch the hoop. | AtN |
| Number of sides | Number of sides making up the shape that is copied by the participant. This applies to the *Coping shapes* activity. | Exact | The shape copied by the participant has the same number of sides as the shape appearing in the model. | NlEx |
| | | Inexact | The shape copied by the participant has a different number of sides as the shape appearing in the model. | Nl |
| Length of sides | Length of the sides making up the shape copied by the participant. This applies to the *Copying shapes* activity. | Adequate | The length of the sides of the shape copied by the participant is equal to that of the sides of the stimulus presented as a model. | LdAd |
| | | Inadequate | The length of the sides of the shape copied by the participant is different from the length of the sides of the stimulus presented as a model. | LdIn |
| Amplitude of angles | Amplitude of the angles (separation distance between the two sides of an angle) making up the shape copied by the participant. This applies to the *Copying shapes* activity. | Appropriate | The amplitude of the angles of the shape copied by the participant is equal to the angles of the shape appearing as a model. | AaAp |
| | | Inappropriate | The amplitude of the angles of the shape copied by the participant is different from the angles of the shape appearing as a model. | AaAId |
| Intersection | Intersection points or union of the lines making up a stimulus. It applies to the stimuli for the *Copying letters, words and numbers* activities. | Same | The points of intersection or union of the elements making up the stimulus copied by the participants are situated at the same positions as those of the model. | Ii |
| | | Different | The points of intersection or union of the elements making up the stimulus copied by the participants are situated at different positions as those of the model. | Id |
| Legibility | Degree to which the letters, words and numbers that are copied by the participants are legible. This applies to the *Copying letters, words and numbers* activity. | Yes | The letters, words or numbers copied by the participant are legible. That is, they can be easily read since their perception is comfortable, simple and requires little effort. | Ls |
| | | No | The letters, words or numbers copied by the participant are not legible. That is, they cannot be easily read since their perception is not comfortable or simple and requires great effort. | Ln |
| Order | Sequence in which the letters of the words copied by the participants are placed or situated. This applies to the stimuli that are words in the *Copying letters, words and numbers* activity. | Equal | All of the letters making up the word copied by the participant appear in the same order as the word offered as the model. | Oi |
| | | Unequal | One or several letters making up the word copied by the participant appear in a different order from the order of those in the word offered as a model. | Od |
| Directionality | Direction, orientation of letters, words or numbers copied by the participant. This applies to all of the stimuli of the *Copying letters, words and numbers* activity. | Correct | The letters, words or numbers copied by the participant have the correct direction/orientation. | Dc |
| | | Incorrect | The letters, words or numbers copied by the participant do not have the correct direction/orientation but rather, they are rotated on their vertical axis (with changes from right to left, as if reflected in a mirror; e.g.: b-d) or on their horizontal axis (with changes above and below; e.g.: b-p). In the case of words, this implies that at least some of their letters have this incorrect directionality. | Di |
